# Systematic Study of Binding of *µ*-Conotoxins to the Sodium Channel Na_V_1.4

**DOI:** 10.3390/toxins6123454

**Published:** 2014-12-18

**Authors:** Somayeh Mahdavi, Serdar Kuyucak

**Affiliations:** School of Physics, University of Sydney, Sydney, New South Wales 2006, Australia; E-Mail: Sahar@physics.usyd.edu.au

**Keywords:** sodium channels, conotoxins, homology modeling, docking, molecular dynamics, potential of mean force, binding free energy

## Abstract

Voltage-gated sodium channels (Na_V_) are fundamental components of the nervous system. Their dysfunction is implicated in a number of neurological disorders, such as chronic pain, making them potential targets for the treatment of such disorders. The prominence of the Na_V_ channels in the nervous system has been exploited by venomous animals for preying purposes, which have developed toxins that can block the Na_V_ channels, thereby disabling their function. Because of their potency, such toxins could provide drug leads for the treatment of neurological disorders associated with Na_V_ channels. However, most toxins lack selectivity for a given target Na_V_ channel, and improving their selectivity profile among the Na_V_1 isoforms is essential for their development as drug leads. Computational methods will be very useful in the solution of such design problems, provided accurate models of the protein-ligand complex can be constructed. Using docking and molecular dynamics simulations, we have recently constructed a model for the Na_V_1.4-*µ*-conotoxin-GIIIA complex and validated it with the ample mutational data available for this complex. Here, we use the validated Na_V_1.4 model in a systematic study of binding other *µ*-conotoxins (PIIIA, KIIIA and BuIIIB) to Na_V_1.4. The binding mode obtained for each complex is shown to be consistent with the available mutation data and binding constants. We compare the binding modes of PIIIA, KIIIA and BuIIIB to that of GIIIA and point out the similarities and differences among them. The detailed information about Na_V_1.4-*µ*-conotoxin interactions provided here will be useful in the design of new Na_V_ channel blocking peptides.

## 1. Introduction

Voltage-gated sodium (Na_V_) channels are membrane proteins that are responsible for the excitation of cells in many organs from the nervous system to heart and muscles [1]. Dysfunction of Na_V_ channels is associated with several disorders, such as neuropathic, cardiac and muscular diseases [2]. Blockers of sodium channels from venomous animals, in particular *µ*-conotoxins from cone snails, have been proposed for the treatment of these diseases [3,4,5]. *µ*-conotoxins provide a rich library of peptide blockers of Na_V_ channels, exhibiting a diverse range of affinities for various Na_V_1 isoforms [6]. For example, KIIIA has the highest affinity for Na_V_1.2 among all Na_V_1 isoforms and, hence, could be a candidate for the treatment of neuropathic disorders [7,8]. Similarly, among all *µ*-conotoxins, KIIIA is the most potent blocker of Na_V_1.7, which is a target for the treatment of chronic pain [9]. Likewise, BuIIIB has a higher affinity for Na_V_1.3 and may be developed as an analgesic [5]. In order to develop *µ*-conotoxins further as drug candidates, their affinity and selectivity profiles for the targeted Na_V_1 isoform need to be improved. Several attempts have been made in this direction, focusing in particular on derivatives of KIIIA [10,11,12,13,14,15]. However, in the absence of structures for Na_V_ channels, it has been difficult to make progress.

Before their potential for drug development was recognized, *µ*-conotoxins were used extensively in experimental studies of the pore domain of Na_V_1 channels [16,17,18,19]. For example, there are plenty of mutation data on binding of *µ*-conotoxins to Na_V_1 channels, which could be exploited to improve the affinity and selectivity properties of *µ*-conotoxins targeting a specific Na_V_1 isoform. Unfortunately, due to the complexity of the binding modes, it has been difficult to uniquely interpret such mutation data, so they have been of limited use in solving the design problems posed by *µ*-conotoxins.

The recent determination of the crystal structures for bacterial Na_V_ channels should help to ameliorate this situation [20,21,22,23,24], provided accurate homology models of mammalian Na_V_ channels could be constructed from the bacterial counterparts. This task is not as straightforward as in potassium channels, because in going from bacterial to mammalian Na_V_ channels: (i) the tetrameric symmetry is lost; (ii) the selectivity filter is not conserved; and (iii) there are no good templates for the linker sequences in the turret region, which widely differs among the four domains. Nevertheless, for precisely the same reasons, crystal structures of mammalian Na_V_ channels are unlikely to be solved in the near future, leaving homology modeling as the best alternative for progress. It will be of crucial importance in such quests to make judicious use of the wealth of functional data available on mammalian Na_V_ channels, both to constrain and to validate the constructed homology models. For this reason, initial efforts have focused on modeling of Na_V_1.4 channel, as a great deal of functional data is available for this channel. These studies include binding of tetrodotoxin [25,26] and *µ*-conotoxins [27,28,29] to the Na_V_1.4 channel.

Previously, we constructed a model of Na_V_1.4 using the crystal structure of the bacterial Na_V_Ab channel and studied the binding of *µ*-conotoxin GIIIA to Na_V_1.4 [29]. This choice was motivated bythe fact that Na_V_1.4-GIIIA was the most studied complex experimentally, offering plenty of functional and mutagenesis data for validating the complex model. Here, we use the validated Na_V_1.4 model to present a systematic study of other *µ*-conotoxins that have therapeutic potential, namely, PIIIA [30], KIIIA [31] and BuIIIB [32]. The channel-toxin complexes are created using docking followed by refinement in molecular dynamics (MD) simulations. This method has been used in the description of potassium channel toxins previously and shown to yield accurate complex structures [33,34,35,36,37,38,39]. The Na_V_1.4-*µ*-conotoxin complexes obtained are validated by comparing the binding modes to available mutation data. In the case of PIIIA, we also calculate the binding free energy and compare it to the experimental value. Comparison of the binding modes for PIIIA, KIIIA and BuIIIB to that of GIIIA reveals a common scaffold in binding of the four *µ*-conotoxins to Na_V_1.4. The systematic study of the binding modes of *µ*-conotoxins presented here will be valuable in the design of peptide analogs targeting a specific Na_V_1 isoform with enhanced affinity and selectivity properties.

## 2. Computational Methods

Here, we briefly discuss the computational methods used in obtaining the channel-toxin complex structures and the binding free energies of toxins. For details of the methods, we refer to our previous work on toxin binding to sodium [29] and potassium channels [33,34,35,36,37,38,39].

### 2.1. Structures of Na_V_1.4 and µ-Conotoxins

**Figure 1:**
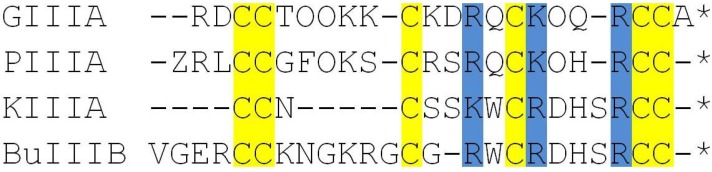
Alignment diagram for the *µ*-conotoxins considered in this study. The Cys residues involved in disulfide bridges are shown in yellow, and the Arg and Lys residues that strongly interact with the channel residues in GIIIA are highlighted in blue. The disulfide bridge pattern for the major isomer is assumed in each case, which is 1–4/2–5/3–6 for PIIIA [40] and BuIIIB [42] and 1–5/2–4/3–6 for KIIIA [41]. The star indicates that the *C*-terminal is amidated.

**Figure 2:**
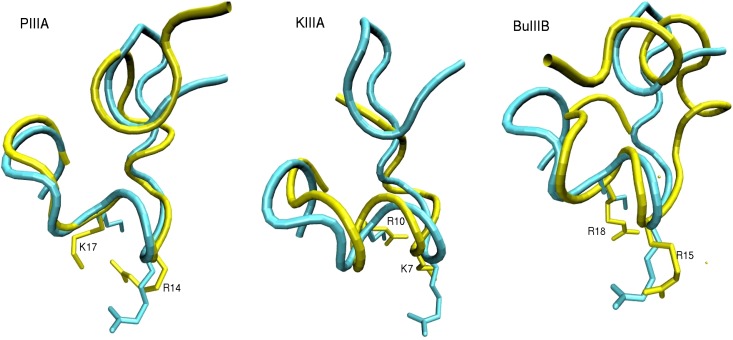
The structures of PIIIA, KIIIA and BuIIIB (yellow) are superposed with that of GIIIA (blue). The side chains of the pore blocking residues R13 and K16 in GIIIA point downward. The corresponding residues in PIIIA (R14 and K17), KIIIA (K7 and R10) and BuIIIB (R15 and R18) are explicitly indicated.

The model for the Na_V_1.4 channel is taken from a previous study [29], where a homology model was constructed using the Na_V_Ab crystal structure [20]. This Na_V_1.4 model was validated by comparing the binding mode and binding free energy results obtained for the Na_V_1.4-GIIIA complex to the available mutagenesis data and the experimental binding free energy [29]. In the following, we will use the binding mode results for the Na_V_1.4-GIIIA complex as a reference in order to facilitate comparison of the binding modes for *µ*-conotoxins PIIIA, KIIIA and BuIIIB,

The alignment diagram for the four *µ*-conotoxins is shown in Figure 1. The three basic residues that are identified as most the significant contributors to the binding of GIIIA to Na_V_1.4 (R13, K16 and R19) are highlighted in blue. To see whether these basic residues are likely to contribute to the binding modes PIIIA, KIIIA and BuIIIB with Na_V_1.4, we superpose their structures with that of GIIIA (Figure 2). The NMR structures of GIIIA [43], PIIIA [44], KIIIA [45] and BuIIIB [42] are taken from the Protein Data Bank with the following respective IDs: 1TCJ, 1R9I, 2LXG and 2LO9. We align the backbone atoms of the C10-Q18 residues in GIIIA with the corresponding ones in PIIIA and KIIIA by minimizing their RMSD. Because there is a gap in BuIIIB at the position of D12, alignment in this case is restricted to the R13-Q18 residues in GIIIA. The R13 and K16 side chains in GIIIA interact with the EEDD ring in Na_V_1.4 and are responsible for the blocking of the pore [29]. We have, therefore, explicitly indicated the R13 and K16 side chains in GIIIA and the corresponding ones in PIIIA, KIIIA and BuIIIB in Figure 2. It is seen that there is a very good overlap between the three *µ*-conotoxins and GIIIA at the binding interface, including the pore blocking R13 and K16 residues in GIIIA. These figures are highly suggestive that all four *µ*-conotoxins may share a common binding motif.

### 2.2. Complex Structures from Docking and MD Simulations

We generate the initial poses for the Na_V_1.4-*µ*-conotoxin complexes using the docking program, HADDOCK [46,47], which has given good results in previous studies of toxin binding to sodium and potassium channels [29,33,34,35,36,37,38,39]. HADDOCK works best when some experimental data are applied as restraints in docking. Unfortunately, unlike GIIIA, there are limited mutagenesis data available for binding of PIIIA and KIIIA to Na_V_1.4 (see Table 1) and none for BuIIIB. We will, therefore, use the binding mode for GIIIA as a guide in docking PIIIA, KIIIA and BuIIIB, which is shown to have very good overlaps with these *µ*-conotoxins at the critical binding interface region (Figure 2). We note that the mutagenesis data in Table 1 is mostly consistent with the binding modes suggested in Figure 2. The large affinity changes arising from the mutation of W8 and H12 in KIIIA are most likely due to misfolding of the toxin rather than loss of any interactions with the channel residues. Thus, we perform two docking calculations for each *µ*-conotoxin, using one of the two pore blocking basic residues identified in Figure 2 and the EEDD ring in Na_V_1.4 as a restraint. The choice of the EEDD ring rather than the DEKA ring is motivated by the fact that the EEDD residues play a much more important role in binding of GIIIA than the DEKA residues [49]. A clustering analysis is performed for the top hundred poses obtained from docking calculations, and a consensus complex is found in each case. Because BuIIIB does not align with GIIIA as well as PIIIA and KIIIA, we have also performed blind docking in this case. The complex obtained from blind docking is found to be consistent with that obtained from restrained docking, confirming that the alignment diagram in Figure 2 is also relevant for the BuIIIB complex.

**Table 1:** Effect of the mutations on the binding affinities of PIIIA and KIIIA to Na_V_1.4. The ratios of the IC_50_ values for the mutated (mut) and wild-type (wt) toxins are given in the third column. The binding constants were determined as 249 nM for PIIIA [48] and 82 nM [11], 48 nM [12] and 37 nM [13] for KIIIA.

Residue	Mutation (Mut.)	$\frac{IC_{50}(mut.)}{IC_{50}(wt)}$	Ref.
**PIIIA**			
R12	A	2.2	[48]
R12	Q	1.6	[48]
R12	K	1.2	[48]
S13	D	6.3	[48]
R14	A	13.0	[48]
R14	Q	15.5	[48]
R14	K	2.2	[48]
K17	A	5.0	[48]
K17	Q	7.0	[48]
H19	Q	0.9	[48]
**KIIIA**			
K7	A	35.7	[13]
K7	A	13.4	[11]
K7	D	18.3	[11]
W8	R	37.3	[12]
W8	Q	73.2	[12]
W8	E	320	[12]
R10	A	27.1	[13]
H12	A	2986	[13]
R14	A	151	[13]

In the next stage, the Na_V_1.4-*µ*-conotoxin complex structure obtained from docking is refined in MD simulations. For this purpose, Na_V_1.4 in the complex structure is aligned with that embedded in the membrane, and the coordinates of *µ*-conotoxin are transferred to the channel model. Following the protocols established for relaxation of ion channels [50,51], the system is equilibrated in several stages. For details, we refer to [29], where the equilibration procedure and the simulation system for the Na_V_1.4-GIIIA complex was discussed in detail. After equilibration, each Na_V_1.4-*µ*-conotoxin complex is simulated for 30 ns to check its stability and to collect data. The trajectory data are then used in the analysis of the binding modes, where the average distances between strongly interacting pairs of atoms at the binding interface are determined.

### 2.3. MD Simulations and Binding Free Energy Calculations

MD simulations are performed using version 2.7 of NAMD [52] with the CHARMM36 force field [53]. An NpT ensemble is used with periodic boundary conditions. Pressure is kept at 1 atm and temperature at 300 K using Langevin coupling with damping coefficients of 5 ps*^−^*^1^. Lennard–Jones interactions are switched off using a smoothing function within a distance of 10–13.5 Å. Electrostatic interactions are calculated using the particle-mesh Ewald algorithm. A time step of 2 fs is employed in MD simulations. The trajectory data is saved at 5-ps intervals, except in umbrella sampling simulations, where the reaction coordinate is saved at every time step.

The binding free energies provide additional means for validation of the complex structures. For this purpose, we construct the potential of mean force (PMF) of the *µ*-conotoxins using umbrella sampling MD simulations, where the toxin is pulled out from the binding pocket to the bulk in small steps. The reaction coordinate is chosen as the distance between the center of mass (COM) of the channel protein and the COM of the toxin along the channel axis. Typically, 30 umbrella windows separated by 0.5 Å are used with a force constant *k* = 30 kcal/mol/Å^2^. Extra windows are inserted if the overlap of densities between two neighboring windows is below 5% or the PMF fails to flatten, which is required to ensure that the bulk region has been reached. The reaction coordinates collected from the simulations are unbiased and combined using the weighted histogram analysis method [54]. Umbrella sampling simulations are continued until the convergence of the PMF is assured from block data analysis of the PMF data.

The binding constant is determined by integrating the PMF, *W* (*z*), along the *z*-axis [33,38]:

(1) $$K_{eq}=\pi R^{2}\int_{z_{1}}^{z_{2}}e^{-W\left(z\right)/k_{B}T}dz$$

where *z*_1_ and *z*_2_ are the initial and final points in the PMF and *πR*^2^ is the average cross-sectional area of the binding pocket, which is determined from the transverse fluctuations of the COM of the toxin. The value of *R*, obtained from restraint-free MD simulations of the Na_V_1.4-PIIIA complex, is 0.64 Å for PIIIA. The standard binding free energy of the toxin is obtained from the binding constant using:

(2) $$G_{b}=-k_{B}T\ln\left(K_{eq}C_{0}\right)$$

where *C*_0_ is the standard concentration of 1 M. Details of the parameters used in umbrella sampling simulations and justification of the 1D approximation used in the determination of the binding constant are given in [33,38].

## 3. Results and Discussion

### 3.1. Binding Modes of the Na_V_1.4-*µ*-Conotoxin Complexes

**Figure 3:**
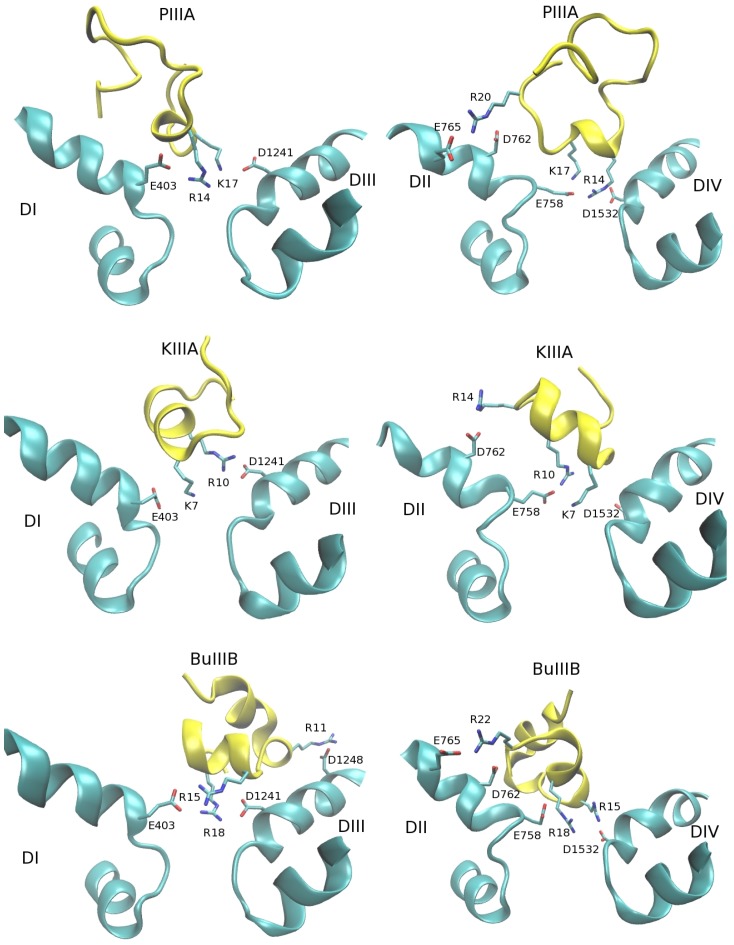
Two views of the Na_V_1.4-*µ*-conotoxin complexes showing the binding mode for domains I and III (**left**) and II and IV (**right**). All of the important interactions between the channel and toxin residues are indicated explicitly.

The docking calculations refined with MD simulations have resulted in fairly unique binding mode for each of the Na_V_1.4-*µ*-conotoxin complexes. Snapshots of the complexes for PIIIA, KIIIA and BuIIIB are shown in Figure 3. In each complex, the basic residues corresponding to R13 and K16 in GIIIA (Figure 1 and Figure 2), namely, R14 and K17 in PIIIA, K7 and R10 in KIIIA and R15 and R18 in BuIIIB, are seen to make contacts with the channel residues, E403, E758, D1241 and D1532, forming the EEDD ring. These interactions clearly dominate the binding mode in each complex. It can be seen from Figure 3 that another common interaction occurs between the basic residue corresponding to R19 in GIIIA, that is, R20 in PIIIA, R14 in KIIIA and R22 in BuIIIB, as well as the D762 and D765 residues in DII. Thus, the Na_V_1.4-*µ*-conotoxin complexes obtained from docking and MD simulations confirm the presence of a common binding motif, involving the three basic residues identified from the alignment diagrams in Figure 1 and Figure 2.

In order to provide a more quantitative description of the complex structures, which is necessary for a detailed comparison of the binding modes, we present in Table 2 the average N–O distances between strongly interacting pairs. The distances are obtained from 30 ns MD simulations of the complex structures. All of the interacting pairs that have an average distance of 4 Å or less have been included in the table. The results for the Na_V_1.4-GIIIA complex [29] are reproduced here, as they provide a useful reference point for comparison. Most of the contact distances are less than 3 Å, indicating a strong coupling between the N and O atoms. Focusing on the interaction of the PIIIA, KIIIA and BuIIIB residues with the EEDD ring first, it is seen that only two basic residues—those identified in Figure 2—are involved in such interactions. The only other common interaction is in between the D762/E765 residues and the third basic residue identified in the alignment diagram (Figure 1). The comprehensive list of the Na_V_1.4-*µ*-conotoxin interactions in Table 2 reinforces the common binding motif proposed from a study of the snapshots in Figure 3.

**Table 2:** List of the interacting residues in the Na_V_1.4-*µ*-conotoxin complexes. The average N–O distances obtained from MD simulations are given for each *µ*-conotoxin complex (in units of Å). The acidic residues, E403, E758, D1241 and D1532, forming the EEDD ring, are listed first, as they are responsible for most of the contacts with the *µ*-conotoxin residues. The D762 and E765 residues in DII provide a second anchoring point for a basic *µ*-conotoxin residue and are listed next.

Na_V_1.4	GIIIA	MD	PIIIA	MD	KIIIA	MD	BuIIIB	MD
E403-O_1_	R13-N_2_	2*.*7	R14-N_1_	2.7	*−*	*−*	R15-N_2_	2.7
E758-O_2_	R13-N_1_	2*.*8	R14-N_1_	2.7	K7-N*_z_*	2*.*9	R18-N_1_	2.7
E758-O_1_	K16-N*_z_*	2*.*7	K17-N*_z_*	2.7	R10-N_1_	2*.*7	R18-N_2_	3.5
D1241-O_2_	K16-N*_z_*	2*.*7	K17-N*_z_*	2.9	R10-N_2_	2*.*8	*−*	*−*
D1241-O_1_	K11-N*_z_*	2*.*7	*−*	*−*	*−*	*−*	R18-N_2_	2.7
D1532-O_1_	K11-N*_z_*	2*.*6	*−*	*−*	*−*	*−*	*−*	
D1532-O_2_	R13-N_2_	2*.*7	R14-N*_E_*	2.9	K7-N*_z_*	3*.*0	R15-N_2_	2*.*7
D762-O_2_	R19-N_2_	2*.*7	R20-N_2_	2.7	R14-N_1_	2*.*7	R22-N_2_	2.7
E765-O_1_	*−*	*−*		*−*	*−*	*−*	R22-N_2_	2.7
D1248-O_1_	K8-N*_z_*	4*.*0	*−*	*−*	*−*	*−*	R11-N_2_	2.8

It is worthwhile to discuss the binding mode for each *µ*-conotoxin complex in light of the proposed common binding motif. In the GIIIA complex, a third basic residue (K11) is engaged with the EEDD ring, which is not seen in other *µ*-conotoxin complexes. In mutagenesis experiments, K11 is found to have the least impact on binding of GIIIA to Na_V_1.4 compared to the residues, R13, K16 and R19, which form the binding motif [55], confirming its secondary role in the binding mode. Another piece of evidence in this regard comes from alanine mutation of K11, namely, GIIIA[K11A] can still block the channel [55]. The channel is blocked when all four acidic residues in the EEDD ring are engaged with basic residues of a toxin [28,29]. It is seen from Table 2 that R13 and K16 residues can still cover all four EEDD residues, and hence, K11 is not needed to block the channel.

A similar binding mode is proposed for the Na_V_1.4-GIIIA complex in [28] with one important difference: R19 was assumed to interact with D1532 as an input in modeling, which was retained in the final result. This was motivated by the observation that the residues corresponding to D762 and R395 in Na_V_Ab make a salt bridge, hence D762 and E765 nearby are not likely to be available for binding of R19. However, there is direct evidence from mutagenesis data that substitutions of D762 and E765 affect the binding of GIIIA [55,56]. Furthermore, there are substantial differences between the Na_V_1.4 and Na_V_Ab structures, which make such an assumption questionable. In our MD simulations of Na_V_1.4, we have not observed the formation of such a salt bridge between D762 and R395. Finally, our MD simulations of the Na_V_1.4-GIIIA complex indicate that the EEDD ring region is not wide enough to accommodate four basic residues. This may have been feasible in the Monte Carlo minimization scheme used in [28], which does not take into account entropic contributions. However, when MD simulations are performed at room temperature for such a configuration, the R19 contact is broken, and it is expelled immediately from the EEDD ring region.

PIIIA exhibits the best alignment with GIIIA and has a very similar affinity for Na_V_1.4, e.g., IC_50_ values are 19 and 36 nM for GIIIA and PIIIA [6]. (In order to facilitate comparisons, we will use the comprehensive set of *µ*-conotoxin affinities provided in [6]). Thus, from the outset, one would expect very similar binding modes for the two complexes, and this is confirmed by the results shown in Table 2. The only notable difference between the GIIIA and PIIIA complexes is the absence of R12 from the binding mode of PIIIA, which corresponds to K11 in GIIIA. This suggests that the maximum number and type of basic residues that the EEDD ring can accommodate is RKK, as in GIIIA, and replacing one of the K residues with the bulkier R residue in this motif as in PIIIA makes it too crowded to fit in the EEDD ring. The loss of one basic residue from the binding mode should result in some loss of affinity in PIIIA relative to GIIIA, which is in line with the quoted IC_50_ values above. The R14 and K17 residues interact with all four EEDD residues (Table 1). Thus, our model predicts blocking of the channel by PIIIA, in agreement with the experimental observations [48].

Our binding mode results for the Na_V_1.4-PIIIA complex again differ from that proposed in [28], where both R12 and R20 were found to make contacts with the EEDD ring. As discussed above for GIIIA, R20 makes contact with the outer D762 residue, and the EEDD ring is not wide enough to accommodate a second arginine on top of R14 and K17, which have established contacts with the EEDD ring. The Na_V_1.4-PIIIA complex was also modeled in [27], where two binding modes with similar binding free energies were proposed. The first involving K9 and R12 is supported neither by the experimental data (Table 1) nor by the arguments based on alignment with GIIIA, which has the best characterized binding mode experimentally. The second binding mode involves R14 and K17, which are fine, but also K9 (instead of R20), which does not occur in our binding mode.

KIIIA is more compact than GIIIA and PIIIA and has only three basic residues. However, it has a good alignment with GIIIA at the binding interface, including the two basic residues (K7 and R10) interacting with the EEDD ring (Figure 2). The main difference is that the RK motif in GIIIA and PIIIA is replaced with KR. Because the lysine side chain is shorter, K7 in that position cannot cover all three domains, leaving E403 free (Table 2). As a result, KIIIA cannot block the channel, in agreement with the experimental observations [13,57]. Loss of another contact in the binding mode of KIIIA relative to PIIIA is expected to reduce the affinity of KIIIA further, which is consistent with the measured IC_50_ value of 90 nM [6].

The Na_V_1.4-KIIIA model proposed in [28] has even more substantial differences from ours compared to the those of GIIIA and PIIIA. This is again driven by use of the D1532-R14 interaction as a constraint in modeling, which appears to have displaced K7 from the EEDD ring. As will be shown in the next section, interaction of an arginine residue with D762 is very strong. Sacrifice of D762-R14 in preference for its interaction with the EEDD ring is not justified, especially if this results in loss of a contact for another basic residue (K7).

BuIIIB also has a good alignment with GIIIA at the binding interface with some small differences, e.g., the RK motif in GIIIA and PIIIA is replaced with RR, the third basic residue (R22) is displaced by one residue and K9 and K11 in GIIIA are replaced with R11 and G14. The R15 and R18 residues make contact with all four EEDD residues (Table 2), making sure that BuIIIB blocks the channel, as observed in experiments [32]. Displacement of R22 enables it to make contact with E765 in addition to D762. The D1248-K8 interaction Na_V_1.4-GIIIA is seen to be relatively weak. Thanks to the longer side chain of arginine, the corresponding D1248-R11 interaction in BuIIIB is much stronger. These more than make up for the loss of a basic interaction (K11 in GIIIA) and could explain the higher affinity of BuIIIB relative to GIIIA (IC_50_ of BuIIIB is 3.6 nM [6]).

### 3.2. Binding Free Energy of PIIIA

**Figure 4:**
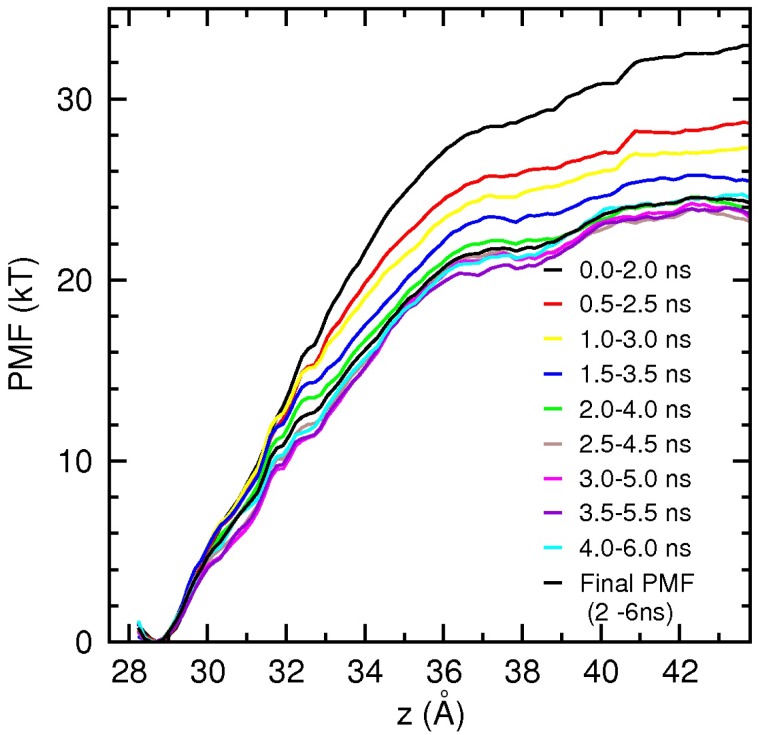
Convergence study of the Na_V_1.4-PIIIA potential of mean force (PMF) from block data analysis. To minimize fluctuations, a large sampling size of 2 ns is used, which is slid in 0.5 ns steps over the available data. The block-data PMFs drop monotonically in the first 2 ns as the system equilibrates and then fluctuate around a baseline, signaling equilibration. The final PMFs obtained from 2 to 6 ns are indicated by a thick black line.

Determination of the binding free energies from the PMF calculations is computationally expensive and laborious. Therefore, we present only the results for dissociation of PIIIA from Na_V_1.4 here, which provide further validation for the proposed binding motif of *µ*-conotoxins. The PIIIA PMF is constructed using umbrella sampling simulations, as discussed in the ComputationalMethods section. PIIIA has a more stable structure compared to GIIIA, and hence, distortion of the toxin during PMF calculations has not been an issue [29,58]. The results of the PMF calculations are presented in Figure 4. To check the convergence of the results and to separate the production data from equilibration, we perform block data analysis of the data. PMFs are constructed from 2 ns blocks of data to reduce fluctuations, and the blocks are slid in 0.5-ns steps over the collected 6 ns of data. The monotonic drop of the PMFs during the first 2 ns indicates that the system is still equilibrating. After 2 ns, the PMFs fluctuate around the baseline, signaling that the system has been equilibrated. The binding constant is determined by integrating the final PMF in Figure 4 using the prescription in Equation (1), and the binding free energy is obtained from Equation 2 as *−*10*.*6 kcal/mol. This is in good agreement with the experimental value of *−*10*.*2 kcal/mol [6], providing further validation for the Na_V_1.4-PIIIA model.

**Figure 5:**
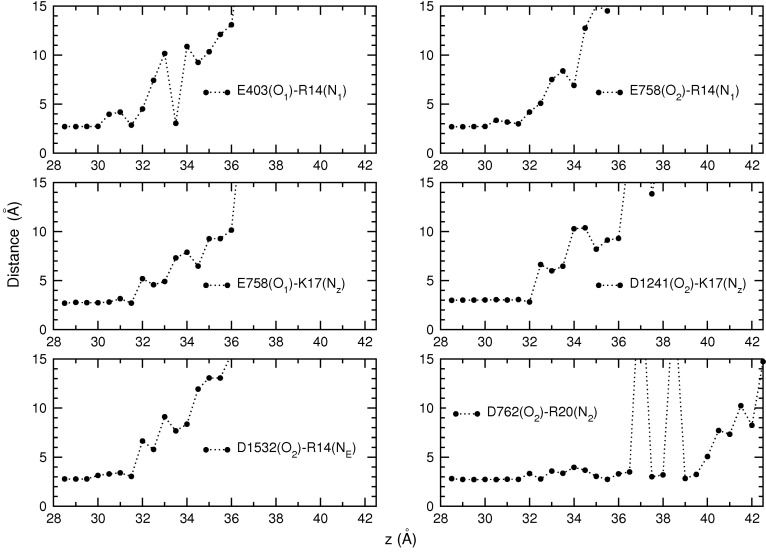
Evolution of the distances between the interacting pairs as PIIIA dissociates from Na_V_1.4. Average N–O distances obtained from each umbrella window are plotted as a function of the window position. All of the pairs listed in Table 2 are considered in the figure.

Umbrella sampling simulations provide a wealth of data on the dissociation process, which can be used to gain further insight into the binding mechanism. A very useful quantity in this regard is the persistence length of the interacting pairs, which roughly gives the distance of the toxin from the binding pocket at which a contact is broken. The persistence length is directly related to the interaction strength; hence, it provides complementary information on the relative strength of the individual interactions in a binding mode. To this end, we have calculated the average N–O distance for each pair in Table 2 using the data from each umbrella window. The calculated distances are plotted as a function of the window position in Figure 5. Inspection of the diagrams in Figure 5 shows that the R14 and K17 residues keep their contact with the EEDD ring up to 32 Å, corresponding to a persistence length of 4 Å. The R20 residue keeps contact with D762 up to 36.5 Å, corresponding to a persistence length of over 8 Å. This indicates that the D762-R20 interaction makes a significant contribution to the binding of PIIIA and provides further justification for including the third arginine residue in the common binding motif proposed in the last subsection.

Variation of contact distances can also be used to understand specific features of the PMF. For example,the initial sharp rise in the PMF up to 32 Å is due to stretching of the toxin in order to maintain its charge contacts with the channel residues. Between 32 and 36 Å, R14 and K17 gradually dissociate from EEDD, while the D762-R20 contact persists, resulting in the less steep rise in the PMF. After 36 Å, all of the contacts are broken, and there is only the long-distance Coulomb interaction between the channel and toxin residues. This results in the slowly rising shoulder region in the PMF between 36 and 41 Å. After 41 Å, the Coulomb interactions are also screened, and the PMF becomes flat.

## 4. Conclusions

We have presented a systematic study of the binding of *µ*-conotoxins to the Na_V_1.4 channel, which revealed a common binding motif for the interaction of *µ*-conotoxins with sodium channels. The binding motif consists of two basic residues (RK or RR) that make contact with the EEDD ring of residues in the pore and a third basic residue (R) that interacts with the higher-lying Asp/Glu residues in domain II (D762/E765 in Na_V_1.4). This is very different from the binding motif found for the toxins that block the potassium channels, which consist of a functional dyad of a pore inserting lysine and an associated aromatic residue, surrounded by a ring of basic residues. Because of the larger vestibule in sodium channels, a single basic residue cannot block the channel. Blocking of the channel is possible when all of the side chains of the EEDD ring are engaged with the basic residues of a toxin, which requires at least two basic residues. As in the case of KIIIA, this may not be enough if the basic residues are not correctly ordered and cannot engage all four EEDD. Another difference from the potassium channels is the lack of tetrameric symmetry. There are few acidic residues in the pore periphery of Na_V_1.4 for anchoring basic residues, e.g., D762/E765 in DII and D1248 in DIII, which is further up and less accessible. Thus, it is not surprising that *µ*-conotoxins have exploited the most available acidic residues in their binding motif.

The binding motifs proposed for GIIIA, PIIIA, KIIIA and BuIIIB provide a general framework for understanding how *µ*-conotoxins interact with sodium channels and will be useful in studies of other *µ*-conotoxins. There are also ongoing efforts for development of drugs from *µ*-conotoxins to treat various diseases associated with dysfunctional sodium channels. The detailed description of the binding modes for the Na_V_1.4-*µ*-conotoxin complexes presented here and in [29] will provide valuable guidance in the design of analogs of *µ*-conotoxins with improved affinity and selectivity properties.
